# Violence exposure and cyberbullying among Chinese adolescents: the mediating role of moral disengagement

**DOI:** 10.3389/fpsyg.2026.1811559

**Published:** 2026-06-18

**Authors:** Xiao Wang, Baoren Liang, Xiaokai Xia, Jie Yin

**Affiliations:** School of Education Sciences, Hengyang Normal University, Hengyang, China

**Keywords:** Chinese adolescents, cyberbullying, mediation, moral disengagement, violence exposure

## Abstract

**Introduction:**

Cyberbullying among adolescents has become a global concern. While existing research confirms a positive association between violence exposure and cyberbullying, the underlying mechanisms, particularly within Chinese adolescent populations, remain poorly understood. This study aims to investigate the mediating role of moral disengagement in the relationship between violence exposure and cyberbullying among Chinese middle school students.

**Methods:**

A cross-sectional survey was administered to 936 middle school students (Grades 7–12) from urban areas in Hunan Province, China. The Violence Exposure Questionnaire, the Moral Disengagement Scale, and the Cyberbullying Questionnaire were employed. Structural equation modeling (SEM) was used to analyze the data and test the mediation model while controlling for demographic variables.

**Results:**

The results indicated that non-only children reported higher levels of violence exposure than only children, and males scored higher on moral disengagement than females. Older adolescents engaged in more cyberbullying. Violence exposure was significantly and positively associated with adolescent cyberbullying. Furthermore, moral disengagement partially mediated this relationship.

**Discussion:**

This study confirms that violence exposure is a significant predictor of cyberbullying and highlights the crucial mediating mechanism of moral disengagement. These findings extend previous research by providing empirical evidence from a Chinese context and suggest that interventions targeting moral disengagement may be effective in reducing cyberbullying perpetration among adolescents.

## 1 Introduction

Cyberbullying among adolescents has become a topic of global concern, with significant regional variations in its prevalence. The reported prevalence rate among Chinese adolescents is approximately 23% (Hong et al., 2024), which is consistent with earlier findings (Li et al., 2019). Cyberbullying is generally defined as repeated, intentional hostile behavior carried out through digital media (Slonje et al., 2013). Common forms include verbal abuse in online forums, mocking comments, and the public disclosure of private conversations (Brewer and Kerslake, 2015). It differs fundamentally from traditional bullying. Due to the anonymity and rapid dissemination inherent in online interactions, cyberbullies can operate beyond temporal and spatial constraints, often inflicting lasting harm on victims (Li, 2007; Englander et al., 2017). Current research predominantly focuses on its negative consequences. High rates of cyberbullying are associated with increased loneliness, depressive symptoms, psychological distress, and suicidal ideation among victims (Hinduja and Patchin, 2010; Iranzo et al., 2019). Furthermore, cyberbullying also severely impacts the mental health of perpetrators and bystanders (John et al., 2018; DeSmet et al., 2019). Given the prevalence and detrimental effects of adolescent cyberbullying, it is crucial to investigate its underlying causes and mechanisms.

Based on social learning theory and the general aggression model, adolescents who actively or passively encounter violent information or events may demonstrate increased acceptance of violence and reinforced aggressive behavioral tendencies (Davis, 2001; Allen and Anderson, 2017). In this study, violence exposure is defined as the violent cues that individuals see, hear, encounter, or experience in their daily environment (Butcher et al., 2015). Adolescents are frequently exposed to such violent cues across multiple contexts. For instance, domestic violence not only predicts involvement in traditional bullying (Mrug and Windle, 2010), but also positively correlated with cyberbullying (Akpunne et al., 2020; Bashir Shaikh et al., 2020). Moreover, with the deep integration of the internet into adolescents’ lives, their opportunities for online engagement have increased significantly (China Internet Network Information Center, 2024). Research has found a positive correlation between frequency of internet use and cyberbullying (Kokkinos and Antoniadou, 2019). Greater time spent playing violent online games is associated with a higher likelihood of involvement in cyberbullying (Zhu et al., 2020; Li et al., 2023), and exposure to violent media exerts a long-term amplifying effect on cyberbullying behaviors (Den Hamer and Konijn, 2015). Together, these findings indicate that adolescents are regularly exposed to violent stimuli in their daily lives, and such exposure is consistently linked to cyberbullying perpetration.

However, although existing research has confirmed a positive association between violence exposure and cyberbullying, the underlying mechanisms of this relationship remain poorly understood. Moral disengagement refers to a psychological process through which individuals selectively deactivate internal moral constraints using cognitive strategies such as moral rationalization and displacement of responsibility. This process serves to reduce feelings of guilt over immoral conduct while allowing individuals to maintain a positive self-image (Heffernan, 1988; Bandura et al., 1996). Social cognitive theory posits that environmental factors can indirectly influence behavioral outcomes by shaping moral cognitive processes (Bandura, 1978). Triggered by violent contexts, moral disengagement is correlated with negative behaviors like bullying and can act as a mediating variable to explain the mechanisms behind such behaviors (Moore, 2015). Previous research has found that moral disengagement may mediate the relationships between community violence, trait anxiety, and cyberbullying (Wang et al., 2017; Esposito et al., 2022). More importantly, adolescents who have experienced early maltreatment are more likely to justify cyberbullying behaviors through moral disengagement (Wang et al., 2019). Therefore, this study proposes that moral disengagement may mediate the relationship between violence exposure and cyberbullying among Chinese adolescents.

Two strands of evidence support this perspective. First, multiple studies indicate that violence exposure correlates with moral disengagement. According to the General Aggression Model, violent situational factors can promote aggressive behavior by influencing an individual’s perception of aggression, negative affect, and physiological arousal (Yao et al., 2019). In real-world contexts, witnessing or experiencing domestic violence has been shown to significantly elevate children’s levels of moral disengagement (Bautista-Aranda et al., 2023). In online contexts, playing violent video games may alter adolescents’ moral standards and can trigger moral disengagement (Gabbiadini et al., 2012; Grizzard et al., 2017; Teng et al., 2017). Furthermore, research on Chinese adolescents indicates that violence exposure positively predicts moral disengagement levels 6 months later (Teng et al., 2019). Second, moral disengagement is a significant predictor of cyberbullying (Bussey et al., 2015; Runions and Bak, 2015; Zhao and Yu, 2021). A previous meta-analysis identified moral disengagement as a major risk factor for perpetrating cyberbullying (Chen et al., 2017). Consistent with this, a longitudinal study demonstrated that moral disengagement significantly predicted adolescent cyberbullying 1 year later (Marín-López et al., 2020).

**Figure 1:**
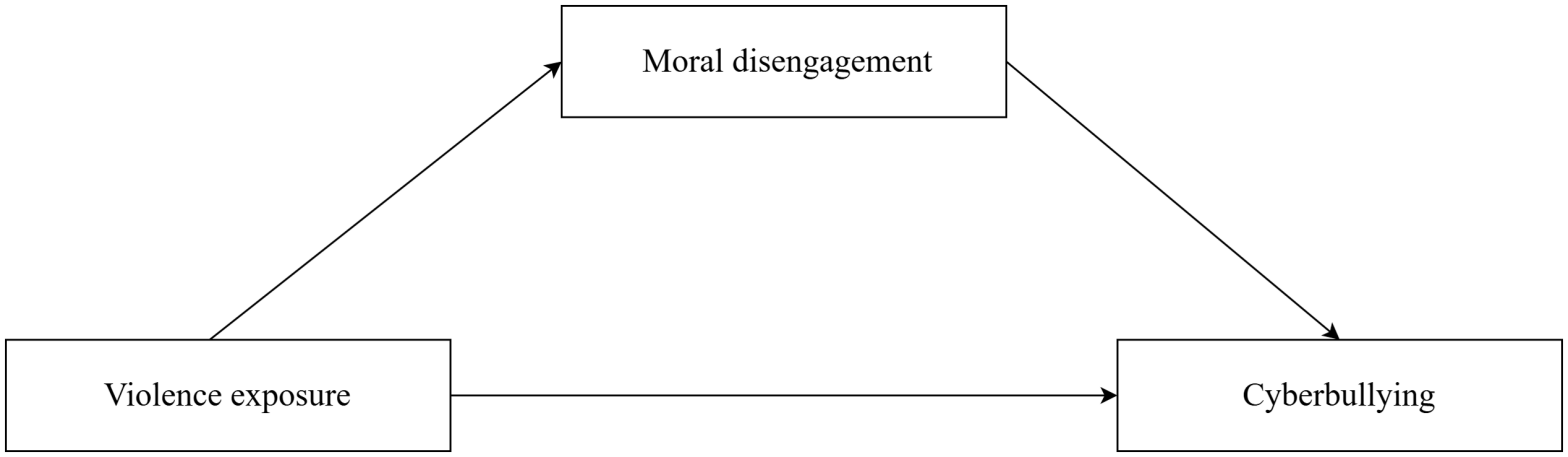
Mediating model of moral disengagement between violence exposure and adolescent cyberbullying.

Despite these advances, existing evidence remains limited in two important ways. First, most prior studies have focused only on the pairwise relationships among violence exposure, moral disengagement, and cyberbullying, with very few examining the interrelationships among all three variables. Second, research specifically targeting Chinese adolescents is particularly scarce. Therefore, as an incremental extension of previous work, the present study aims to replicate and extend the mediation model in a sample of Chinese middle school students (see Figure 1), with a particular focus on cyberbullying perpetration. Specifically, the following hypotheses are proposed:

*H1*: Violence exposure, moral disengagement, and adolescent cyberbullying are positively correlated.

*H2*: Moral disengagement mediates the relationship between violence exposure and adolescent cyberbullying.

## 2 Materials and methods

### 2.1 Participants

This study selecting 1,000 secondary school students (grades 7 to 12) from three urban middle schools in Loudi City and Yongzhou City, Hunan Province, China. The Violence Exposure Questionnaire, Moral Disengagement Scale, and Cyberbullying Scale were administered by class. After excluding invalid questionnaires with obvious errors or excessive missing data, 936 valid responses were obtained, resulting in a valid response rate of 93.6%. The sample consisted of 457 females and 479 males, aged 11 to 19 years (mean age = 14.42 years, standard deviation = 1.73 years). Among the participants, 11.2% were from only-child families.

### 2.2 Instruments

#### 2.2.1 Violence exposure

The Violence Exposure Questionnaire developed by Schwartz and Proctor was employed to assess adolescents’ violence exposure (Schwartz and Proctor, 2000). The scale comprises 25 items, each rated on a 5-point Likert scale ranging from 1 (*never*) to 5 (*always*). Higher scores indicate that individuals are more likely to encounter violent cues in their daily lives and thus have a higher level of violence exposure. The Chinese version of this scale has demonstrated good reliability and validity in adolescent samples (Zhang et al., 2021). In the present study, it showed excellent internal consistency, with a Cronbach’s alpha coefficient of 0.94.

#### 2.2.2 Moral disengagement

The Moral Disengagement Scale developed by Bandura was employed to assess adolescents’ levels of moral disengagement (Bandura et al., 1996). The scale comprises eight subscales, each containing four items, for a total of 32 items. These subscales are moral justification, euphemistic labeling, advantageous comparison, displacement of personal responsibility, diffusion of personal responsibility, distortion of consequences, attribution of blame, and dehumanization of victims. All items were rated on a 5-point Likert scale ranging from 1 (*strongly disagree*) to 5 (*strongly agree*). The mean score across all items was calculated, with higher scores indicating a greater propensity for moral disengagement. The Chinese version of this scale has demonstrated good reliability and validity among adolescents (Wang et al., 2017). In the current study, it exhibited good internal consistency, with a Cronbach’s alpha of 0.82.

#### 2.2.3 Cyberbullying

The Cyberbullying Scale developed by Wright was employed to assess adolescents’ online bullying behaviors (Wright, 2014). This scale comprises nine items, each rated on a 5-point Likert scale ranging from 1 (*never*) to 5 (*always*). The mean score across all items was computed, with higher scores indicating a higher frequency of cyberbullying perpetration. The Chinese version of this scale has demonstrated good reliability and validity in adolescent samples (Wang et al., 2021). In this study, it showed excellent internal consistency, with a Cronbach’s alpha of 0.91.

### 2.3 Procedure

This study was reviewed and approved by the Research Ethics Committee of Hengyang Normal University. To recruit participants, we adopted a convenience cluster sampling approach. Three urban public middle schools in Loudi City and Yongzhou City, Hunan Province, China, were selected based on geographic accessibility and willingness to participate. Within each school, classes served as natural clusters, and all students in grades 7 to 12 from the selected classes were invited to take part. Trained research assistants administered paper-based questionnaires to the students in their regular classrooms during school hours. Before completing the questionnaires, all participants provided both written and verbal informed consent. They were assured of the anonymity of their responses and that the data would be used solely for research purposes. Demographic information, including age, gender, grade, and only-child status, was also collected. A total of 1,000 secondary school students were initially recruited, and all completed the survey under the supervision of the research assistants.

### 2.4 Data analysis

We screened the collected questionnaires for invalid responses prior to data analysis. Invalid questionnaires were defined as those meeting any of the following criteria (Curran, 2016; Howell, 2007): (a) more than 50% of the items on any scale were missing; (b) the same response option was selected for 20 or more consecutive items; (c) the standard deviation of responses across the last 30 items was zero; or (d) there were obvious contradictory or patterned responses indicating careless responding. Using SPSS 23.0, we identified and excluded invalid cases based on these prespecified criteria. Because detailed raw data before screening were not retained, we are unable to report the exact frequency of each exclusion reason. However, after removing invalid questionnaires, 936 valid responses remained out of the initial 1,000 participants, yielding a valid response rate of 93.6%.

This study employed a cross-sectional survey design with questionnaire-based data collection, which carries the risk of common method variance. To assess this, Harman’s single-factor test was conducted. The analysis showed that all 12 factors had eigenvalues greater than 1. The first factor explained 21.67% of the variance, which is below the critical threshold of 40%. Therefore, the data were deemed reliable, with no significant issue of common method bias. Subsequently, SPSS 23.0 was used to perform independent-samples t-tests and one-way ANOVA on demographic variables and to calculate descriptive statistics and Pearson correlation coefficients for all study variables. To examine the mediating role of moral disengagement, a structural equation model was constructed using AMOS 28.0 (Collier, 2020). After controlling for variables such as gender and grade, the total effect of violence exposure on cyberbullying was tested first. Moral disengagement was then added to the model as a mediator. The significance of the indirect effect was tested using the bootstrap method with 5,000 resamples, employing bias-corrected confidence intervals to determine its significance.

## 3 Results

### 3.1 Preliminary analyses

Independent-samples *t*-tests revealed no significant gender differences in violence exposure or cyberbullying among adolescents. However, a significant gender difference was found in moral disengagement (*t* = 3.56, *p* < 0.001), with male students scoring significantly higher than female students. Additionally, a significant difference was observed between only children and non-only children in violence exposure (*t* = 3.15, *p* < 0.01), with non-only children reporting higher scores. The means, standard deviations, and correlation coefficients for all variables are presented in Table 1. Significant correlations were found among violence exposure, moral disengagement, and cyberbullying. Specifically, violence exposure was positively correlated with moral disengagement, which in turn was positively correlated with cyberbullying; violence exposure was also directly and positively correlated with cyberbullying. Furthermore, one-way ANOVA results indicated significant grade-level differences in violence exposure (*F* = 7.91, *p* < 0.001), cyberbullying (*F* = 5.30, *p* < 0.001), and moral disengagement (*F* = 22.14, *p* < 0.001). Post-hoc comparisons revealed that ninth- and twelfth-grade students scored significantly higher on these three variables than their peers in other grades within the same educational stage (see Table 2).

**Table 1:** Mean, standard deviation, and correlation coefficient among variables.

Variable	M	SD	1	2	3
1. Violence exposure	40.91	13.93	–		
2. Moral disengagement	61.62	15.74	0.34**	–	
3. Cyberbullying	16.94	3.62	0.35**	0.40**	–

*N* = 986. ^*^p < 0.05, ^**^p < 0.01.

**Table 2:** Grade-level differences in adolescent’s violence exposure, moral disengagement, and cyberbullying.

Variable	1 (*N* = 216)	2 (*N* = 180)	3 (*N* = 158)	4 (*N* = 148)	5 (*N* = 135)	6 (*N* = 99)	*F*	*LSD*
1. Violence exposure	37.49 ± 11.41	40.59 ± 14.55	40.11 ± 12.64	40.47 ± 14.50	43.87 ± 15.92	46.89 ± 13.65	7.91***	1 < 2,3,4 < 5,6
2. Moral disengagement	54.30 ± 14.02	59.24 ± 16.06	64.09 ± 14.42	61.75 ± 15.26	67.81 ± 14.88	69.34 ± 14.98	22.14***	1 < 2 < 3,4 < 5,6
3. Cyberbullying	16.63 ± 3.29	16.67 ± 3.48	16.55 ± 3.08	16.55 ± 3.14	17.76 ± 3.88	18.24 ± 4.98	5.30***	1,2,3,4 < 5,6

1 = 7th grade, 2 = 8th grade, 3 = 9th grade, 4 = 10th grade, 5 = 11th grade, 6 = 12th grade. ***p < 0.001.

### 3.2 Testing for the mediation effect

This study employed AMOS 28.0 to construct latent variable models to examine the mediating role of moral disengagement in the relationship between violence exposure and cyberbullying among adolescents. First, a total effect model was tested with violence exposure as the predictor and cyberbullying as the outcome variable. This model demonstrated a good fit (χ^2^/df = 0.82, RMSEA = 0.04, CFI = 0.98, GFI = 0.97), with violence exposure was significantly associated with cyberbullying (*β* = 0.48, *p* < 0.001).

Second, a mediation model incorporating moral disengagement was tested. Given the unidimensional nature of the violence exposure and cyberbullying measures, items for violence exposure were parceled into three indicators using the factorial algorithm method, and items for cyberbullying were randomly assigned to three parcels (Wu and Wen, 2011; Little et al., 2022). The fit indices indicated that the mediation model had a good fit (see Table 3).

**Table 3:** Intermediate model fitting metrics table.

χ2	df	χ2/*df*	RMSEA	GFI	AGFI	IFI	RFI	TLI	CFI	NFI
194.95	74	2.64	0.04	0.97	0.96	0.98	0.97	0.98	0.98	0.97

**Figure 2:**
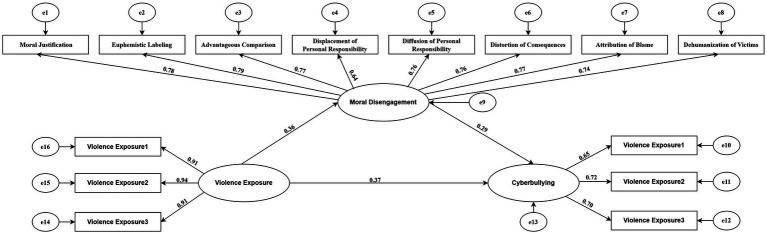
Model of the relationship between violence exposure, moral disengagement, and adolescent cyberbullying.

The significance of the path coefficients was then examined. As shown in Figure 2, violence exposure had a significant positive effect on moral disengagement (Path a: *β* = 0.36, *p* < 0.001), moral disengagement significantly and positively predicted cyberbullying (Path b: *β* = 0.29, *p* < 0.001), and violence exposure retained a significant direct positive effect on cyberbullying (Path c’: *β* = 0.37, *p* < 0.001). Using the bootstrap method with 5,000 resamples, the bias-corrected confidence interval for the indirect effect (ab) was [0.02, 0.03], which does not include zero.

First, a total effects model was constructed with violent exposure as the predictor variable and cyberbullying as the outcome variable. The model showed a good fit to the data: χ^2^/df = 0.82, RMSEA = 0.04, CFI = 0.98, GFI = 0.97. Violence exposure was significantly associated with cyberbullying (*β* = 0.48, *p* < 0.001). This result indicates that moral disengagement partially mediated the relationship between violence exposure and adolescent cyberbullying. The direct effect accounted for 77% of the total effect, while the indirect effect accounted for 23% (see Table 4).

**Table 4:** Decomposition table of total effects, direct effects, and mediating effects of moral disengagement.

Effect	Standardized estimates	Nonstandard estimate	BootSE	Effect proportion	Bias-corrected 95%CI		
					Lower	Upper	*p*
Direct effect	0.37	0.09	0.01	77%	0.06	0.11	***
Mediation effect(ab)	0.11	0.02	0.00	23%	0.02	0.03	***
Total effect(c)	0.48	0.11	0.01		0.09	0.14	***

^***^p < 0.001.

## 4 Discussion

This cross-sectional study investigated the associations among violence exposure, moral disengagement, and cyberbullying in a sample of Chinese adolescents. The findings indicated that non-only-children reported higher levels of violence exposure than only-children, male students scored higher on moral disengagement than females, and students in terminal grades (like ninth and twelfth grades) scored higher on all three variables compared to their peers in lower grades. After controlling for gender and grade, violence exposure was positively associated with cyberbullying, and this association was partially mediated by moral disengagement. These results are consistent with theoretical expectations, but given the cross-sectional design, they should be interpreted as statistical associations rather than causal pathways.

### 4.1 Characteristics of adolescent’s violence exposure, moral disengagement, and the development of cyberbullying

The refinement of campus violence prevention regulations and increasingly stringent enforcement of laws protecting adolescents have effectively reduced adolescents’ exposure to violent incidents (Jiang et al., 2019). In the current study, adolescents who were non-only children reported significantly higher levels of violence exposure. This disparity might be tentatively explained by differences in family environment. In our sample, the majority of adolescents (88.8%) had siblings. Previous research has shown that non-only children are more likely to experience sibling bullying (Wolke and Samara, 2004), and that sibling interactions often involve more verbal and physical aggression than peer interactions (Duncan, 1999). Although we did not directly measure sibling bullying, it is plausible that such dynamics contribute to the higher violence exposure scores observed among non-only children in this sample.

This study also revealed significant gender differences in adolescents’ levels of moral disengagement. Prevailing gender roles often socialize males to exhibit greater competitiveness and aggression, while emphasizing emotional empathy and relationship maintenance for females. Moral disengagement is a self-regulatory mechanism that enables individuals to engage in behaviors contravening their moral standards without experiencing distress (Bandura, 2002). Prior research has indicated that males are more likely to employ moral disengagement to rationalize aggressive acts (Bussey et al., 2015). Consequently, when engaging in aggression, males may more readily invoke such cognitive strategies to mitigate feelings of guilt, which may account for their higher observed levels of moral disengagement. This gender difference is consistent with previous findings indicating that male adolescents are more likely to employ moral disengagement mechanisms than females to justify aggressive behaviors (Caroli and Sagone, 2014).

Additionally, this study found that students in the higher grades of a school (i.e., ninth and twelfth grades) exhibited higher levels of violence exposure, moral disengagement, and cyberbullying. As students progress through their education, the social environments they encounter become more complex, which may increase their likelihood of encountering violent stimuli. According to the General Aggression Model, violence exposure within specific contexts can exert a latent influence on aggressive behavior (Allen and Anderson, 2017). Concurrently, with the proliferation of smart devices, older adolescents often turn to the internet to cope with academic pressure. The online environment contains substantial violent content, and research has shown that repeated exposure to violent media and video games is significantly associated with a higher incidence of cyberbullying among adolescents (Den Hamer and Konijn, 2015; McInroy, 2017; Zhu et al., 2020). The operation of any moral standard inherently involves the potential for moral disengagement (Bandura et al., 1996). Given that cyberbullying constitutes a form of online immoral conduct (Meter and Bauman, 2018), older students who engage in it may invoke moral disengagement mechanisms to rationalize their actions, thereby mitigating self-blame.

### 4.2 The association between violence exposure and adolescent cyberbullying

The positive correlation between violence exposure and cyberbullying is consistent with the direction predicted by stress theory, which posits that chronic stressors such as violence exposure may be associated with increased aggressive responses (Al-Badayneh et al., 2024). Similarly, prior research has suggested that prolonged exposure to violent contexts could be linked to desensitization to aggression (Manchikanti Gómez, 2011). However, because our data are cross-sectional, we cannot determine whether violence exposure directly leads to desensitization or cyberbullying; these theoretical pathways remain plausible but require longitudinal testing.

This study found that violence exposure was not only directly associated with adolescent cyberbullying but also indirectly linked to it through moral disengagement. Furthermore, the stability of moral disengagement is a critical concern. Longitudinal evidence suggests that adolescents who maintain high levels of moral disengagement are at a greater risk of exhibiting persistent aggressive and violent behaviors in late adolescence (Paciello et al., 2008). According to the theory of triadic reciprocal determinism, violent environmental factors may influence an individual’s moral judgments via social negative mechanisms, potentially promoting moral disengagement (Bandura, 1978; Bandura et al., 1996). Previous theoretical and empirical work has suggested that violence exposure could relate to moral disengagement through pathways such as cognitive restructuring, emotional desensitization, and social learning (Grizzard et al., 2017; Allen et al., 2022; Bautista-Aranda et al., 2023). Consistently, the General Aggression Model proposes that prolonged exposure to violent situations may heighten moral disengagement and thereby reinforce aggressive tendencies (Allen and Anderson, 2017).

Importantly, our cross-sectional mediation analysis can only demonstrate that the indirect path is statistically consistent with these theoretical accounts, but it cannot establish the causal ordering or confirm the specific mechanisms. For example, although moral disengagement is theorized to weaken self-regulatory control and make cyberbullying appear more acceptable (Meter and Bauman, 2018), we cannot rule out reverse causality—adolescents who already engage in cyberbullying might develop higher moral disengagement over time. Additionally, the amplifying effect of online anonymity on moral disengagement has been discussed in prior literature (Khan et al., 2020), but our data do not directly test this contextual modulation. Future longitudinal or experimental studies are needed to examine whether the indirect pathway operates as theorized.

## 5 Limitations and practical implications

This study has several limitations. First, the reliance on self-report measures may introduce biases such as social desirability and common method variance. Future research could incorporate interviews or experimental designs to mitigate these concerns. Second, we adopted a convenience cluster sampling approach, and all participants were recruited from three urban middle schools in two cities in Huan Province, China. This regional and urban-only sample limits the generalizability of the findings to other populations, such as rural adolescents or student form other provinces. Third, the cross-sectional design cannot establish temporal precedence or infer causality among violence exposure, moral disengagement, and cyberbullying. Although the tested mediation model is theoretically grounded, the indirect paths should be interpreted as statistical associations consistent with the proposed framework, not as causal mechanisms. Alternative explanations, such as reverse causality or unmeasured confounding, remain possible. Future longitudinal or experimental studies are needed to examine the directional nature of these relationships. Additionally, integrating intervention research would help validate the current finfings and provide stronger evidence to inform practice.

Despite its limitations, this study confirms the relationship between adolescent violence exposure and cyberbullying and examines the mediating role of moral disengagement. Notably, this study extends previous research by examining the mediating role of moral disengagement in a Chinese middle school sample while simultaneously controlling for key demographic variables such as gender, grade, and sibling status. Investigating the underlying mechanisms of this relationship contributes to and enriches the existing literature. Furthermore, this study has significant practical implications. First, it found that students in terminal grades and non-only children face higher risks of violence exposure and cyberbullying, indicating a need for targeted interventions. For students in higher grades, anti-violence education tailored to their cognitive level should be developed, utilizing case studies to illustrate the serious consequences of aggressive behavior. For families with multiple children, greater attention should be paid to family interaction patterns. Second, home-school collaboration should be strengthened. Parents are encouraged to foster a positive home environment and monitor their children’s exposure to violent content. For families in special circumstances (e.g., single-parent or left-behind families), schools should provide additional family education guidance. Such guidance can help adolescents understand the real-world impact of their online behavior and enhance their sense of moral responsibility.

## 6 Conclusion

This study reveals significant grade-level differences in adolescents’ violence exposure, cyberbullying, and moral disengagement. Given the well-documented negative impacts of both violence exposure and cyberbullying on adolescent development, reducing exposure to violent factors at the school and family levels remains crucial. Moreover, interventions aimed at mitigating cyberbullying may benefit from targeting moral disengagement. However, these conclusions are drawn from a cross-sectional sample of urban adolescents in Hunan Province, China, and should be replicated in broader populations before firm recommendations are made.

## Data Availability

The original contributions presented in the study are included in the article/supplementary material, further inquiries can be directed to the corresponding author.
